# The Application of VR Technology in Engineering Issues: Geodesy and Geomatics, Mining, Environmental Protection and Occupational Safety

**DOI:** 10.3390/s25226848

**Published:** 2025-11-09

**Authors:** Paweł Strzałkowski, Kinga Romańczukiewicz, Paweł Bęś, Barbara Delijewska, Magdalena Sitarska, Mateusz Janiszewski

**Affiliations:** 1 Faculty of Geoengineering, Mining and Geology, Wrocław University of Science and Technology, Wybrzeże Wyspiańskiego 27, 50-370 Wrocław, Poland; kinga.romanczukiewicz@pwr.edu.pl (K.R.); pawel.bes@pwr.edu.pl (P.B.); barbara.delijewska@pwr.edu.pl (B.D.); magdalena.sitarska@pwr.edu.pl (M.S.); 2 Department of Civil Engineering, School of Engineering, Aalto University, FI-00076 Aalto, Finland; mateusz.janiszewski@aalto.fi

**Keywords:** virtual reality, VR, immersive technology, engineering, sensors

## Abstract

Sensors are a key component of virtual reality (VR) technology, as they enable motion tracking, interaction with the environment, and realistic representation of user behaviour in virtual space. VR technology is gaining increasing importance in engineering, offering new ways to support research, analysis, and training. This article examines its applications in four key areas: surveying and geomatics, mining, environmental protection, and occupational safety. The study is based on a review of the scientific literature indexed in the Scopus database, with the aim of highlighting both the potential of VR and directions for its future development. The findings indicate that VR provides effective tools for analyzing, interpreting, and visualizing complex geospatial data. It enables realistic simulations of mining processes, supports the monitoring of environmental impacts, and facilitates environmental education by creating engaging, immersive experiences. In occupational safety, VR allows hazard scenarios and accident events to be reproduced in a safe yet highly realistic environment, significantly enhancing the effectiveness of training. This is made possible through the integration of sensors with virtual reality, further enhancing immersion in the environment. Despite these advantages, several barriers have been identified. They include technological challenges, insufficient numbers of trained specialists, health and ergonomics concerns, resistance to organizational change, ethical considerations, and limited funding. It is clear that the future of VR in engineering will be shaped by continuous technological progress combined with growing attention to behavioural aspects of training and user interaction. These trends are expected to drive the creation of increasingly advanced and effective tools. The article thus provides a foundation for further exploration of VR as an integral part of engineering practice.

## 1. Introduction

Virtual reality (VR) is a technology that enables the creation of interactive, immersive three-dimensional environments using computers, where users can perform tasks and experience situations resembling real-world conditions. Specifically, users can interact with these environments through various devices, such as VR headsets, which may evoke the sensation of being present in a physically absent yet seemingly real space [1,2]. Since its inception, VR has undergone significant evolution, starting with simple simulations in the mid-20th century, progressing through the development of advanced technologies in the 1990s, and extending to contemporary applications in design, education, and training [3,4]. Over the years, VR has become not only a tool for the entertainment industry but also an essential component of scientific research, where its application contributes to improving the efficiency of educational and analytical processes [5,6,7]. It is currently also applied in therapy and work optimization. Despite its wide-ranging potential, VR still requires extensive research not only on the possibilities of its application but also on potential adverse effects [7,8].

In recent years, there has been a notable increase in research interest regarding VR applications across various scientific fields. This trend has been driven by the availability of low-cost VR devices and their enhanced performance, which has opened new research and educational opportunities [9]. In the field of engineering sciences, VR is used for simulating complex technical processes and training. Examples include applications in mechanical engineering, construction, mining, and geodesy, where the technology facilitates the understanding of complex systems and processes through the creation of realistic models [5,10,11]. In particular, VR represents an excellent tool for supporting education and assessing acquired skills, thereby enhancing the quality of technical education [6]. This enables students and professionals to gain experience in a safe environment, eliminating risks associated with mistakes in real-world conditions [12,13].

**Figure 1:**
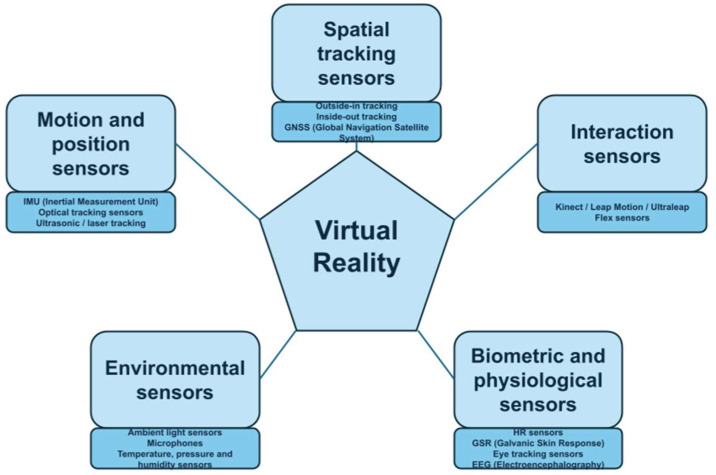
VR and cooperating sensors.

Experiences and real sensory perceptions evoke stronger emotional reactions (affective responses) compared to so-called substitute experiences, such as images or videos. However, it is not always possible to be physically present in a given environment and directly perceive environmental stimuli. In such cases, virtual reality proves extremely useful, as it induces directed behaviour of the organism through artificial sensory simulation. This effect can be achieved either through computer-generated environments or through pre-recorded and replayed videos [8]. Currently, VR designers create rich, computer-generated 3D environments that allow participants to perceive visual and auditory stimuli as if they were real. Head-mounted displays digitally track head movements in physical space, creating depth perception and rendering different images for each eye. Additionally, such displays isolate the user from the external world, producing the sensation of being immersed in a three-dimensional virtual environment [8,14]. VR technology functions in close conjunction with sensors (Figure 1), which possess a variety of functions and serve to enhance the user’s immersion in the virtual environment, thereby engendering the sensation of being transferred to an alternate reality. A variety of sensor technologies form the basis of functional VR systems, enabling real-time tracking, feedback and localisation in various engineering applications. These technologies include inertial measurement units (IMUs), physiological sensors (e.g., heart rate sensors) and GNSS modules. On the other hand, sensors provide data on human behaviour when using VR and human responses to various content presented in the virtual world. Consequently, sensors represent a valuable instrument in evaluating the effectiveness and relevance of content presented in VR.

As VR technologies become increasingly accessible, their application in scientific research is becoming more diverse. Contemporary studies suggest that VR can enhance learning efficiency and knowledge acquisition, as demonstrated by numerous scientific investigations [15,16]. In particular, VR assists in engaging participants and providing them with interactive experiences, which facilitates a deeper understanding of the processes under study. The immersive nature of VR engages participants’ multisensory perceptions, enabling more effective learning and acquisition of practical skills [5,13,17]. Compared with traditional teaching methods, VR transforms the way knowledge is acquired, leading to a better understanding of complex issues and more active involvement in learning materials [4,18,19].

The use of virtual reality in the field of geosciences has been extensively explored by numerous researchers and is primarily focused on improving efficiency, safety, and innovation in technological and organizational processes. As a technology that enables immersive simulations, VR has the potential to revolutionize training methods, design processes, and risk management. Examples of VR applications in mining include simulations of working conditions in mines, which allow workers to acquire skills in a safe environment and to test safety procedures prior to their implementation in real situations [2,20,21,22]. In geodesy, VR facilitates the visualization of data and the analysis of spatial models. Surveying tools are also employed to construct spatial models for the purpose of developing simulation environments and VR scenarios [23,24]. In the field of environmental protection, VR is used to create simulations aimed at educating society about the consequences of climate change and human activity on ecosystems [14,25,26]. In the domain of occupational safety and health, VR plays a pivotal role in training employees, teaching them procedures for responding to hazardous situations, and minimizing the risk of workplace accidents [2,27,28,29].

The aim of this article is to provide a literature review on the applications of virtual reality technology in geodesy and geomatics, mining, environmental protection, and occupational safety. The present article aims to identify both common and sector-specific challenges related to the implementation of VR, with particular emphasis on the role and requirements for the integration of sensors that enable realistic representation of environmental, spatial and operational conditions. The current state of research on the utilization of VR in these domains underscores its capacity for generating innovative solutions and delineates avenues for prospective future applications. Moreover, the research findings furnish information on the limitations of VR use and the possibilities of integrating virtual reality technology with sensors. This integration allows for the identification of potential research directions for improving the accuracy, immersion, and effectiveness of VR applications in the discussed areas of engineering. This article serves as a starting point for further exploration and research in the context of integrating virtual reality into engineering practice.

## 2. Materials and Methods

**Figure 2:**
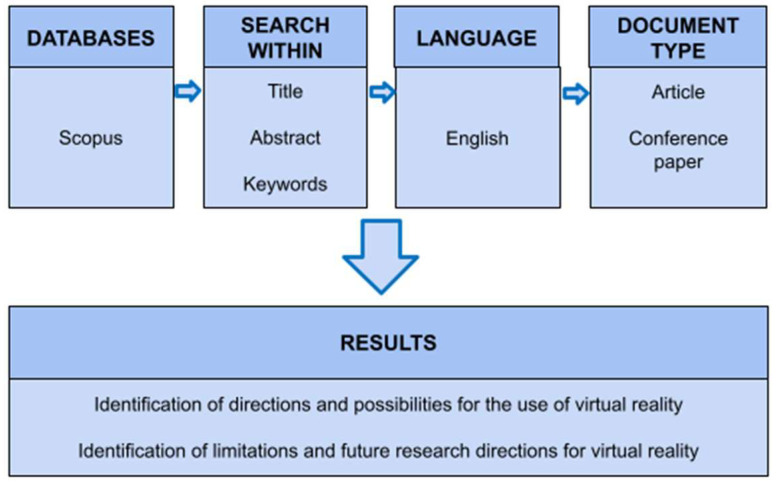
Diagram of the methodology of the conducted research.

The Scopus database was searched for studies on the application of VR technology in the fields of mining, geodesy, environmental engineering, and occupational safety. The following criteria were applied: original articles or conference papers published in English in peer-reviewed journals, with no restrictions on the year of publication. The adopted methodology for conducting the literature review is presented in Figure 2.

**Figure 3:**
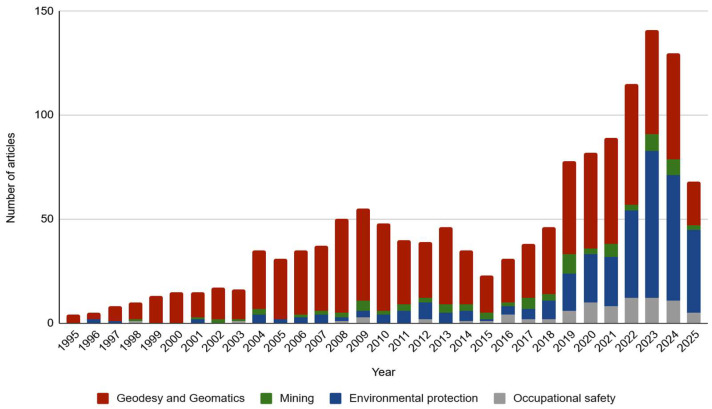
The number of literature results in specific years.

The source analysis was carried out based on predefined keywords (Table 1). As a result, a total of 3332 records were obtained. However, due to thematic inconsistency (e.g., not relating to the engineering areas discussed or incorrect meaning of keywords) or brief thematic mention in a significant number of publications, only those relevant to the subject of this article were selected. In total, 1395 records were retained. The analysis demonstrated a growing interest in VR, with scholarly attention to the use of virtual reality in the areas covered by this article beginning in 1995 (Figure 3). The increasing number of publications reflects the development and rising popularity of VR technology as well as its applications in various domains, including geodesy, mining, environmental protection, and occupational safety.

## 3. VR in Engineering Topics

### 3.1. VR in Geodesy and Geomatics

Geodesy is an interdisciplinary field of science and engineering concerned with determining the shape, dimensions, and location of objects in space, both on the Earth’s surface and beneath it. It encompasses methods of field measurement, ranging from classical angular and linear observations to advanced satellite techniques, laser scanning, and approaches to processing and visualizing spatial data. Modern geodesy extends beyond its measurement functions, playing a key role in spatial planning, environmental monitoring, cultural heritage documentation, engineering design, and infrastructure management. Referring to the full spectrum of methods and applications, the term geomatics is increasingly used, as it represents a domain at the intersection of geodesy, cartography, topography, and computer science. Geomatics can be defined as “a systemic, multidisciplinary, integrated approach to selecting instruments and appropriate techniques for collecting, storing, integrating, modelling, analyzing, querying as needed, transforming, displaying, and distributing spatially georeferenced data from various sources with strictly defined accuracy and continuity parameters in digital format” [30,31].

An integral part of contemporary geodesy and geomatics is spatial modelling, understood as a set of methods and techniques used to process and transform measurement data into a digital representation of reality. From a technical perspective, this involves the creation of three-dimensional models representing objects and structures, both those located on the surface and underground, as well as the representation of terrain relief, land cover, and elements of cultural and natural landscapes. In the process of measuring and gathering information on the geometry of objects, geomatics most often employs two complementary approaches: LiDAR-based methods and image-based methods that rely on the analysis of photographs [32,33,34,35,36]. Complementing the classification by measurement methods, an equally important division in geomatics relates to the type of measurement platform used, as this influences the range, mobility, and accuracy of the measurement. Three main groups can be distinguished: TLS (Terrestrial Laser Scanning) [37], MMS (Mobile Mapping Systems) [38], and UAS (Unmanned Aerial Systems) [39].

In addition to methods based on direct spatial data acquisition, an important supplement to the 3D modelling process is the reconstruction of objects from drawings, including both precise technical projects and conceptual sketches. This approach is known as parametric modelling and originates primarily from disciplines such as industrial design [40], computer graphics, and architecture [41]. It is also applied in geomatics, especially in cases where measurement data are unavailable and reconstruction relies on archival documentation. Three-dimensional models can be created from orthogonal projections, structural cross-sections, or perspective sketches using geometric methods or AI-assisted techniques. Although this method is subject to limitations arising from the absence of real spatial data, it may serve as a valuable supplement in projects related to the reconstruction of historical objects [41], the gaming industry [42], or VR visualizations [43].

The approaches and methods described above are crucial in the process of spatial modelling. Their diversity makes it possible to adapt them to the specific characteristics of the environment being represented, as well as to requirements regarding scale, accuracy, and data presentation format. They form the foundation and a toolkit for subsequent applications in virtual reality, supporting various areas of use: from education and spatial planning to heritage preservation and communication with the end user.

Whereas Section 3.1.1 addressed the methodological and technical foundations of spatial data acquisition, Section 3.1.2 focuses on the analytical and practical implications of integrating such geomatic datasets into virtual reality systems.

#### 3.1.1. Geometry Acquisition and Reconstruction Approaches for VR

LiDAR (Light Detection and Ranging) technology can operate based on different principles of distance measurement. One of them is the Time-of-Flight (ToF) method, which relies on measuring the time required for a laser pulse to travel from the emitter to the object and back to the detector. Another approach is phase-based measurement, which analyses the phase shift between the transmitted and reflected waves, enabling precise depth determination. The third method is triangulation-based, in which the distance is calculated on the basis of known geometric relationships between the source, reflection point, and detector [44,45].

In addition to recording spatial coordinates, LiDAR systems also store reflection intensity values, providing supplementary information about the reflective properties of scanned surfaces. Various types of laser scanners are available on the market, differing in parameters such as measurement range, accuracy, resolution, laser wavelength, field of view, and scanning speed, as well as in design characteristics including size, weight, mobility, and the ability to integrate with other systems (e.g., GNSS, IMU, cameras) [46,47]. The performance of each type of sensor depends on its optical configuration, range and signal characteristics, which together determine the achievable measurement accuracy of the data for VR integration. Short-range triangulation scanners offer sub-millimetre precision and are particularly suitable for detailed documentation of small objects, architectural or industrial components. However, their high accuracy comes with limited spatial range and sensitivity to surface colour and material. Phase scanners represent an intermediate class, offering millimetre accuracy at high scanning speeds, making them well suited for building interiors, archaeological sites, and cultural heritage preservation tasks. Long-range ToF systems, on the other hand, are capable of acquiring data from distances of several hundred metres, making them indispensable for measuring large areas and engineering structures, although they typically have lower point density and reduced spatial resolution, which makes them indispensable for surveying large terrains and engineering structures. The optical properties of the emitted laser beam also play a key role. The wavelength of the laser, typically between 532 nm (green) and 1550 nm (infrared), determines how the signal interacts with different surface materials [48,49]. Shorter wavelengths (green) provide higher reflectivity on bright or smooth surfaces but are more susceptible to atmospheric scattering, while near-infrared beams penetrate vegetation and atmospheric haze more effectively [50]. The choice of the appropriate sensor configuration is therefore a compromise between the required accuracy, range and compatibility with visual datasets.

These sensor configurations directly affect how 3D data is later integrated into VR environments. For example, scanners equipped with IMU sensors and GNSS receivers provide precise georeferencing of point clouds, while RGB or multispectral cameras mounted together enable photorealistic rendering by combining geometry with colour and texture information [51]. This fusion of sensors is fundamental to ensuring that virtual reconstructions retain both geometric accuracy and visual realism [52,53].

In the context of 3D modelling for VR integration, LiDAR technology is gaining importance as one of the most accurate tools for acquiring spatial data. Thanks to the high density of point clouds, it is possible to create highly detailed models of object geometry and entire scenes with millimetre-level accuracy, using both Terrestrial Laser Scanning (TLS) and Airborne Laser Scanning (ALS). Such models, in the form of point clouds, after processing, e.g., generating a triangular mesh and applying textures, become the basis for constructing VR environments. At the same time, this technology is not without limitations. In some cases, the high cost of equipment and time-consuming scanning, large dataset sizes, and the necessity of processing (e.g., point cloud filtering, mesh reconstruction, texturing) may pose barriers for certain projects. For large-scale projects, especially those involving complex or covered structures, the data acquisition process often requires the use of multiple scanning stations to ensure complete coverage of the area. This not only increases field time, but also computational requirements during point cloud registration and alignment. The resulting datasets can exceed several billion points, requiring data reduction and segmentation processes before they can be used for VR integration. Furthermore, LiDAR does not record colour information without integration with other instruments such as RGB cameras, which requires additional imaging to obtain a fully photorealistic visualization [54].

Each of these platforms is applied in different spatial and project conditions. TLS enables precise scanning of architectural, industrial, and geological objects in high resolution. It is used, among others, in heritage documentation, engineering structure measurements, deformation analysis, or geotechnical hazard monitoring [55]. ALS, on the other hand, due to its ability to rapidly acquire data from high altitudes, is effective in mapping extensive areas, both urbanized and natural, e.g., in terrain morphology analyses, land cover change assessment, watershed modelling, or landslide hazard evaluation. In the VR context, ALS data can serve to build extensive 3D landscapes, where the user can analyze space from a pedestrian or aerial perspective, monitor erosional processes, or design infrastructural changes [56]. An example application may include virtual reconstruction of areas degraded by mining, where the researcher analyses potential reclamation scenarios in an interactive 3D environment. Mobile Mapping Systems (MMS), as an intermediate technology, offer a compromise between TLS precision and ALS mobility, enabling fast mapping of linear infrastructure (e.g., roads, railways, mining corridors) as well as indoor spaces of buildings or industrial facilities. Mobile systems are particularly useful where traditional stationary scanning would be too time-consuming and ALS data insufficiently detailed [44].

Despite numerous advantages of LiDAR technology, such as high geometric accuracy and the ability to record large areas in a short time, it does not always constitute the only or optimal source of spatial data for creating 3D models for VR environments. In many cases, photogrammetric methods provide an alternative or complement to laser scanning, allowing object geometry reconstruction based on image analysis. This method, relying on single photographs or sets of images taken from different viewpoints, makes it possible to reconstruct the three-dimensional geometry of objects and obtain realistic surface textures [57]. Of particular importance in this process are Structure-from-Motion (SfM) and Multi-View Stereo (MVS) algorithms, which enable automatic reproduction of the spatial arrangement of points (point cloud) and the creation of a dense mesh model with applied textures representing the real appearance and colours of the object [58]. SfM, combined with MVS, is a computer vision–based method enabling 3D model reconstruction using images only. Unlike classical stereophotogrammetry, SfM allows the derivation of 3D information from overlapping images without prior knowledge of the camera’s position, orientation, or calibration, and without the need to establish ground control points. This enables the use of low-cost imaging systems, both in aerial and terrestrial applications [59,60]. In the literature, photogrammetric methods are categorized, among others, by the image acquisition approach [61]. The most common division includes the following [59,62]:

– Aerial photogrammetry, where images are acquired from an aircraft or an Unmanned Aerial Vehicle (UAV), usually in nadir mode, for cartographic, environmental, or spatial landscape analyses.
– Terrestrial photogrammetry, based on images captured from ground level using cameras on tripods or handheld, widely applied in façade documentation, architectural details, and engineering structures.
– Close-range photogrammetry, involving images taken from short distances (up to several metres), used in engineering, archaeology, heritage conservation, or technical inspections. It can be carried out both from ground level and using UAVs at low altitudes.

Each of these methods differs in application scope, spatial resolution, technical requirements, and integration potential with geodetic systems and VR environments. Aerial photogrammetry enables rapid data acquisition for large areas but is limited in resolution and vertical object detail. Close-range photogrammetry provides highly detailed models and textures but requires suitable lighting conditions, numerous images, and precise calibration or georeferencing [63,64]. The achievable accuracy of photogrammetric reconstructions depends on ground sampling distance (GSD), image overlap, and camera calibration stability. Typical UAV-based photogrammetry achieves horizontal accuracies of 2–5 cm, whereas terrestrial close-range imaging can reach sub-centimetre precision under controlled conditions. However, the method is sensitive to variations in illumination and object texture, which can significantly affect the reliability of surface reconstruction and texture mapping in VR applications [65,66].

In geodesy, photogrammetry is used, among others, for building inventory, documentation of engineering and architectural structures, and quality assessment of construction works [65,67]. Three-dimensional models derived from images can be georeferenced using geodetic markers or GNSS data, allowing their further use in measurement, design or documentation tasks. In VR environments, the same models may serve as virtual replicas of reality, enabling spatial analysis, technical condition inspection, or simulation of engineering processes without the need for physical presence at the site.

In image-based methods, sensor properties such as focal length, pixel size and dynamic range control the achievable resolution and colour depth of textures used in VR. Combined with GNSS and IMU data, optical sensors enable precise orientation and alignment with LiDAR-based datasets, resulting in hybrid 3D models that combine geometric accuracy with visual realism [68].

One of the key advantages of photogrammetry in the context of VR applications is its wide technological accessibility and relatively low hardware and skill entry threshold. By using standard digital cameras, including those integrated with UAVs, it is possible to acquire image data in diverse terrain and spatial conditions without the need for specialized scanners [32]. Photogrammetry allows simultaneous reproduction of geometry and texture of objects, resulting in 3D models with a high level of visual realism. Such models are particularly valuable in VR environments designed to deliver immersive user experiences. Examples include virtual reconstructions of cultural heritage, digital visualizations of archaeological [69] and historical monuments [70], as well as applications in cultural tourism [71,72]. Another advantage of the method is its scalability. Photogrammetry can be successfully applied both for the reproduction of small architectural or engineering elements and for modelling larger terrain structures, such as sections of spoil heaps, ruins, slopes, or archaeological sites [73].

The process of creating models ready for use in virtual reality based on geospatial data involves several consecutive stages and depends on the chosen method of data acquisition. First, raw point clouds or image sets are processed through registration and georeferencing, which allows them to be aligned in a consistent spatial reference frame. This stage often involves the integration of GNSS/IMU metadata and control points to ensure measurement accuracy. Next, point clouds are filtered, merged and converted into polygonal meshes that describe the geometry of the object. Photogrammetric datasets are reconstructed to obtain dense 3D point sets and surface models. Once the geometry has been determined, texture mapping is performed using calibrated RGB or multispectral images, which increases the visual realism of the scene [74]. In the next phase of optimization, the model is simplified to balance performance and fidelity, using level of detail (LOD) hierarchies or mesh reduction to maintain smooth rendering in the gaming environment. The final stage involves composing the scene in the virtual environment engine, where geometry, textures, lighting and interaction logic [64]. At this stage, accurate sensor data and high-quality pre-processing ensure spatial and visual consistency, which are key to achieving both scientific credibility and user immersion in VR applications.

#### 3.1.2. VR Application in Classical Geodesy and Geomatics

In recent years, a dynamic development of 3D spatial technologies has been observed, with applications increasingly widespread across various fields. Tools enabling three-dimensional modelling, visualization of measurement data, and the integration of spatial information with digital project environments have become an indispensable component of contemporary surveying and engineering processes. In particular, VR technology has been gaining importance as a user interface that allows immersive data exploration, analysis, and support for project and implementation decision-making.

Given the broad thematic scope and the large number of scientific publications addressing the use of 3D tools, including photogrammetry, 3D structure modelling, LiDAR data, visualizations, digital twins, and point cloud processing, the scope of this review was deliberately narrowed. The focus was placed on areas most closely related to classical surveying activities, where VR serves as a tool supporting surveying and design processes.

Additionally, in order to illustrate the broader context of VR in geomatics, selected examples of its applications in cartography and Geographic Information Systems (GIS) are presented. These include interactive exploration of spatial data, education through three-dimensional maps, and support for environmental and urban analyses.

A particularly important area of VR application, consistent with traditional surveying practice, is its use in the context of classical field measurements such as levelling, total station surveying, or GNSS observations. VR enables the simulation of real-world field conditions and measurement processes in a virtual environment, opening new possibilities for education, procedure testing, and survey planning. In educational contexts, VR technology is applied in the development of interactive geodetic laboratories, where users can practice the entire measurement process, from instrument setup to calculations and result interpretation, without the need for physical presence in the field [75,76,77]. An example of this approach is the virtual levelling laboratory developed by Bolkas et al. [78], which allows students to replicate field conditions and perform surveying tasks in line with standard methodology [64]. A similar approach was taken by Qi et al. [79], who designed a VR classroom environment integrated with WebGIS, enabling the simulation of surveying tasks and the real-time exploration of spatial data. VR is also becoming a tool that supports the prior planning of measurement stations in complex terrain conditions, such as infrastructure construction sites, urbanized areas, or hard-to-access locations. By visualizing topography, terrain relief, and obstacles, the surveyor can preliminarily assess visibility between points, optimize measurement routes, and estimate the risk of measurement errors [76]. Dunmoye et al. [80] further emphasized the collaborative aspect, noting that VR environments enhance team interaction, strengthen the sense of presence and increase so-called social presence, i.e., the feeling of co-participation in a task. Bolkas et al. [75] compared the educational effectiveness of VR with educational games, indicating higher levels of engagement and realism achieved through VR technology. Importantly, their study also demonstrated that VR can replicate core engineering surveying procedures, such as levelling loops, with a high degree of realism and measurement precision, achieving misclosures within approximately 1 mm. The authors emphasized that such virtual experiments not only enhance student engagement but also foster a critical understanding of measurement accuracy, error propagation, and methodological correctness. The results demonstrate that VR can emulate real-world surveying conditions with metrological fidelity, supporting the evaluation of measurement precision and the validation of field procedures within a controlled virtual framework. This example demonstrates the analytical potential of VR in surveying, showing its usefulness for training but also for spatial data visualization and evaluation of measurement.

Besides training, VR gives an analytical framework that connects education with real-world practice. By allowing users to “step into” a simulated measurement environment, VR improves spatial perception and situational awareness, which are super important for assessing data quality, evaluating project feasibility, and interpreting research results. It also facilitates collaborative learning and decision-making, as multiple users can explore and discuss the same virtual scene in real time [80].

One of the other key applications of VR technology in surveying and geomatics is the visualization and analysis of measurement data. Visualization and analysis of survey data constitute a fundamental area in which VR adds value to both classical and modern surveying practices. Three-dimensional data acquired from total stations, laser scanning, GNSS measurements, or photogrammetry can be processed and imported into VR-supported environments, where they can be explored and sometimes interpreted. The user can navigate through a terrain or object model, perform cross-sections, measure distances, assess slopes, and detect deviations from the project. Compared to traditional methods of data inspection (e.g., in CAD or 2D GIS environments), VR offers a more intuitive and engaging way of interpreting survey results and 3D models, which is significant for design, inspection, and construction engineering coordination. The ability to directly “enter” the 3D model and view it at full scale enhances the understanding of spatial relationships and facilitates decision-making during project implementation.

An increasing number of studies highlight the growing role of VR in analyzing data acquired from various measurement platforms. In the literature, VR systems are particularly often used for visualization and representation of 3D data structures. Gençtürk et al. [81] described a methodology for integrating photogrammetric 3D city models (from UAV and terrestrial systems) with the Unreal Engine to create immersive VR environments. The authors demonstrated how processed OBJ/MTL data can be imported along with terrain coverage (Cesium World Terrain) and GIS data, enabling realistic urban landscape visualization and large-scale building analysis. Similarly, Pavelka and Landa [82] proposed a comprehensive workflow utilizing QGIS for GIS data preparation (DEM, GeoTIFF, thematic layers), followed by Unreal Engine for visualization and animation of these data in VR/AR. Their study addressed both performance issues in handling large datasets and applications on mobile devices. Their research discussed GPU algorithms and their application in terrain analysis, providing the theoretical basis for implementing such processes in VR environments. In the context of large-area visualization are presented techniques for terrain surface visualization, where LiDAR, photogrammetric and satellite data are used to generate topographic models [83,84].

The use of VR technology and visualization is particularly common in cultural heritage preservation and documentation, employing various 3D data acquisition techniques [85,86]. Such models not only serve as digital archives of the preservation state but also support conservation processes, enable structural analysis, and allow reconstruction of destroyed, inaccessible, or non-existent objects based on archival and historical data. This makes it possible to “recover” heritage that has been physically lost, which is of particular significance in cases of war destruction or natural disasters. An example is the work of Williams et al. [53], in which the authors presented the application of photogrammetry in documenting geoparks and geological sites of cultural and scientific importance. Another example of integrating multiple measurement techniques for archaeological heritage documentation was provided by Calisi et al. [87], where TLS and UAV were combined to develop a detailed 3D model of the Roman archaeological complex of Santa Croce in Gerusalemme, later implemented into a VR environment for exploration and digital conservation.

In this context, the growing role of VR technology as a tool supporting the development of cultural and natural tourism should also be emphasized, both in educational and popularization dimensions. By enabling virtual tours of heritage sites, archaeological excavations, museums, or parks, VR provides access to places that are otherwise geographically or temporally difficult to visit [83,88,89].

The integration of high-resolution spatial data within VR environments transforms visualization into an active process of spatial analysis and decision support. Three-dimensional data visualization in VR allows surveyors and engineers to inspect areas of interest, evaluate spatial relationships and simulate measurement scenarios, which improves the understanding of terrain structure and project feasibility. These functions are particularly useful in tasks such as visibility and line-of-sight studies, volumetric analyses in open-pit mining. In addition to technical advantages, VR provides users with a new analytical dimension. Immersive visualization improves spatial perception, enabling developers to intuitively detect inconsistencies, understand terrain morphology and evaluate alternative designs before implementation [90].

The literature also highlights the increasing importance of integrating VR technology with Building Information Modelling (BIM). The combination of VR and BIM allows the visualization of digital models of construction and infrastructure objects, supporting design, inspection, and operation [91]. From the perspective of surveying and geomatics, VR + BIM facilitates comparison between survey data and project models, supports deviation detection, and enhances coordination and life cycle management of objects. Podkosova et al. [92] presented the BIMFlexi-VR system, which enables integration of parametric building models with immersive VR environments, allowing interactive structural analysis and instant visualization of design changes. Johansson et al. [93] emphasized the significance of VR integration with openBIM in team collaboration, multidisciplinary coordination, and real-time project reviews, demonstrating the potential of VR as a tool supporting engineering cooperation. In the field of classical surveying applications, an interesting example is the Scan-to-HBIM-to-VR approach presented by Tini et al. [94], where TLS and UAV data were used to develop a parametric model of a historic building, subsequently made available in VR for immersive inspection and digital conservation.

The integration of VR and BIM becomes particularly important in the context of the digital twin concept, which connects measurement data and 3D models with monitoring and management processes throughout the entire life cycle of objects, increasingly applied in both engineering and urban practices. During design reviews, VR data integration enables evaluation of projects, where engineers, planners and clients can jointly explore 3D data, identify potential conflicts and discuss modifications in real time. This shift from passive data inspection to active spatial interaction improves the accuracy of analyses and supports decision-making processes.

Despite these advances, several technical and methodological challenges still hinder the broader adoption of VR in geomatics. One of the main difficulties is integrating different spatial data sources, such as LiDAR, photogrammetry, GNSS and BIM, which vary in resolution, coordinate systems and file formats [95]. Transforming these datasets into one optimized 3D model suitable for VR environments often results in a loss of geometric accuracy, texture quality or georeferencing precision, which can affect the analytical credibility of virtual representations. As a result, the metric accuracy and analytical reliability of virtual representations may be compromised, particularly when VR environments are intended not only for visualization but also for quantitative assessment and engineering analysis [96]. Furthermore, the lack of standardized data exchange protocols between geodetic software and VR platforms exacerbates the problem, leading to the need for manual data conversion and increased risk of cumulative transformation errors.

A further obstacle lies in hardware performance and real-time rendering efficiency, which remain critical factors in the scalability of VR for geodetic applications. This is strongly related to the size of the data collected in the field, which often requires high computing power. High-density point clouds and complex mesh models derived from terrestrial or aerial surveys can easily exceed the memory and processing capacity of standard GPUs. To maintain smooth performance, datasets must frequently undergo simplification, downsampling or mesh decimation, processes that inevitably lead to the loss of fine-scale spatial detail or geometric continuity. The balance between visual realism, frame rate stability and metric precision is a constant limitation for analytical VR environments [97].

Therefore, certain common tendencies can be observed in the analyzed examples. Most VR applications in geomatics are still oriented toward visualization rather than analysis, focusing on spatial understanding, education or collaboration rather than quantitative computation. Analytical functionalities remain limited [98].

Altogether, integrating sensor-based spatial data with virtual reality environments is a complex, multi-step process, limited by both technological and methodological factors. The analyzed studies indicate that 3D data with different resolutions, coordinate reference systems and data structures represents a major obstacle to achieving fluent interoperability between VR, GIS and BIM platforms. Additional limitations come from hardware constraints, data volume and the lack of standard protocols. Nevertheless, the examined studies demonstrate that VR is a highly effective tool. Its ability to bridge realism with analytical insight provides engineers and researchers with novel opportunities for design evaluation and measurement validation [64].

### 3.2. VR in Mining

Mining belongs to those industrial sectors where work is associated with a high level of risk, harsh environmental conditions, and the necessity for continuous improvement of technological and organizational processes. One of the tools that has been gaining importance in this context is virtual reality (VR). It enables the creation of interactive digital environments that allow real situations to be simulated under safe and controlled conditions.

This technology is applied not only in training and education, but also in the areas of occupational safety and the design and monitoring of mining processes [2]. Thanks to the possibility of accurately reproducing mining spaces, VR can serve as a valuable tool supporting the sector’s development.

Over the past two decades, modern technologies have advanced rapidly, and VR has gained recognition as a technology that supports the development of the mining industry. One of the most frequently described areas of VR application is training. VR simulations allow workers to practice emergency procedures, navigate mine workings, and operate mining machinery under realistic conditions, which leads to better preparation for real tasks [99]. As a result, the risk and costs can be significantly reduced compared to traditional training methods, while simultaneously increasing learning effectiveness. Studies show that such training reduces risks and costs, while enhancing effectiveness through the immersive nature of the medium [100,101]. The possibility of repeating procedures multiple times without the risk of equipment damage or endangering lives makes VR an exceptionally effective teaching tool [102,103,104,105,106,107]. A good example is VR-based simulators for mining machinery operators, which allow training in controlled environments and show a positive impact on learning outcomes [2].

Education and knowledge dissemination about mining represent another area in which VR is increasingly applied. Preparing future engineers and mining specialists requires training them for work in difficult and dangerous environments that cannot be easily reproduced in a classroom setting. VR offers new opportunities in this regard, enabling the creation of immersive educational experiences that allow students to experience mining realities before descending underground. Through virtual mine tours, it is possible to demonstrate the complexity of mining processes, technical infrastructure, and associated hazards in an engaging yet safe way. Such solutions support both academic teaching and the dissemination of knowledge about mining to the broader public, especially those with no direct access to underground workings. Franchini et al. [108] presented a simulator of a lunar environment developed for educational and experimental purposes. Although designed with space research in mind, the authors note that its mechanisms can be adapted to mining, where limited space, challenging environmental conditions, and high-risk factors are also present. The ability to simulate underground conditions in a safe environment makes VR an invaluable tool in education. Students can practice navigating tunnels, learn safety protocols, and operate machinery before entering real mine workings.

Work safety is a critical challenge in mining, where everyday activities involve natural, technical, technological, human, and organizational hazards [2]. Traditional training, though essential, does not fully convey the dynamics and complexity of such risks. In this context, VR becomes a breakthrough technology, enabling workers to prepare for emergencies in a realistic yet safe manner. Masood and Egger [109] emphasize that VR changes the way humans interact with machines, allowing for better hazard awareness and faster decision-making. Thanks to immersive VR simulations, it is possible to practice emergency procedures and decision-making under time pressure in conditions that accurately replicate the mining environment. Research shows that participants in such training sessions identify hazards faster and respond more effectively than those trained using traditional methods [100,101,110]. Importantly, these effects are long-lasting, Grabowski and Jankowski [111] demonstrated that safety procedures were remembered even several months after completing the course. VR in mining thus functions not only as a training tool, but also as an element that helps shape a safety culture and reduce accident rates.

Designing, simulating, and monitoring mining operations requires consideration of many factors, including geological conditions, safety, and economic efficiency. Traditional planning methods based on schematics or numerical analyses often fail to accurately reflect the complexity of underground workings. In this context, VR gains special significance by offering tools for 3D visualization, simulations, and interactive monitoring [112,113,114,115]. Strzałkowski et al. [2] emphasize that VR enables the creation of digital representations of mines, which support not only the design stage, but also ongoing monitoring and technological decision-making. Virtual mine models allow for the realistic reconstruction of mining conditions and the analysis of different operational scenarios. This includes testing layout designs, simulating ventilation systems, or predicting the effects of alternative technical solutions. Such an approach reduces the risk of design errors while enhancing the transparency of the planning process.

Research by Strzałkowski et al. [2] highlighted that VR is particularly useful in assessing mine stability and identifying areas of increased geotechnical risk. By interacting with the digital environment, users could verify predictions of rock mass behaviour and better understand potential hazards in mining operations. Another example of VR application is its integration with the concept of digital twins. Combining virtual models with real measurement data enables the creation of dynamic mine representations that evolve alongside mining progress. Don et al. [115] indicate that such solutions allow engineers to simulate the consequences of their decisions before implementation, significantly improving safety and efficiency. VR also supports mining operation monitoring through integration with GIS systems and spatial data. This enables multi-layered visualizations that combine geological models with technical infrastructure and equipment parameters. According to Strzałkowski et al. [2], such solutions facilitate not only transport and ventilation planning, but also comprehensive risk analysis.

Nevertheless, further research should examine how immersive safety training can be systematically integrated into existing occupational safety management systems and evaluated through long-term, evidence-based frameworks.

Although VR has demonstrated considerable potential in mining design, training, and safety management, several technical and organizational limitations continue to restrict its wider adoption. A recurring challenge concerns the integration of heterogeneous spatial and sensor data, such as LiDAR point clouds, photogrammetric models, and geotechnical monitoring data into coherent, real-time VR environments. Differences in spatial resolution, coordinate systems, and temporal frequency often lead to misalignments or inconsistencies that reduce the reliability of simulations [112,113]. Sensor latency and limited accuracy in extreme underground conditions, such as restricted GNSS coverage or dust interference, can further degrade system performance [2].

Different technological approaches show distinct trade-offs. LiDAR-based digital twins provide high geometric precision and are particularly valuable for stability assessment, yet they generate large datasets that demand significant computational power and optimized rendering pipelines [114]. Photogrammetric reconstructions offer greater visual realism but are sensitive to illumination and surface reflectivity, which can be problematic in low-light mining environments. Hybrid approaches combining LiDAR, photogrammetry, and IoT sensor streams offer promising results but raise challenges in data fusion, calibration, and long-term synchronization [115].

In addition to sensor-related issues, immersive mining simulations face difficulties in maintaining real-time performance while handling complex underground geometries and dynamic environmental conditions. High hardware requirements, interoperability gaps between simulation software and mine monitoring systems, and the absence of standardized data exchange protocols hinder seamless integration into daily operations [2,115]. Addressing these challenges, through improved data fusion frameworks, standardized interoperability architectures, and adaptive rendering algorithms, will be crucial for transforming current VR mining applications from isolated research tools into reliable, scalable components of digital mine management systems.

### 3.3. VR in Environmental Protection

One of the greatest global challenges faced by humanity is climate change. This process began many years ago and has led to dramatic environmental consequences that are currently being experienced in many parts of the world. Moreover, in recent years these effects have intensified in areas where they were previously observed and have started to appear in new regions where they had not been noticeable before. However, not everyone is aware of climate change, and many find it difficult to imagine its consequences. The level of awareness and concern among people regarding climate change does not reflect the actual scale of the problem. Scientific research has demonstrated that VR is a suitable tool for visualizing these changes and enhancing their understanding. For many, climate change remains an abstract concept due to both temporal and spatial distance. Temporal distance refers to the fact that actions taken many years ago are only now producing effects, while those undertaken today will only manifest in the future, affecting new generations. Spatial distance means that the consequences of emissions do not always occur where pollutants are released, but rather in completely different locations across the globe. Using traditional means of communication, such as films, images in brochures, or websites, it is possible to provide effective environmental education [116] and visualize climate change [14]. Nevertheless, only by offering a realistic experience with psychological impact can the message be fully internalized [8,117]. For this reason, VR technology is increasingly being used as a tool for pro-environmental education, and its short- and long-term effects are compared with traditional methods of teaching and influencing social opinions and behaviours [118,119]. Some argue that excessive reliance on technology may hinder learning [120], but such opinions are far less frequent than those highlighting its usefulness [25,121,122].

To foster desired pro-environmental behaviours, it is first necessary to raise awareness of the problems that need to be solved. Increasing public awareness and encouraging discussion with others in society are therefore crucial. This was examined by Meijers et al. [118], who investigated how immersive media experiences (VR) affect the willingness to engage in discussions on climate change compared to traditional text-based methods such as articles. Their study found that participants who were presented with climate change scenarios through VR were more willing and more frequent in engaging in such discussions than those who were only exposed to written scientific publications. Moreover, the time span following the presentation during which participants raised the topic was longer in the VR group compared to the traditional group. These findings suggest that visually engaging representations of climate change can increase social interest in the subject [118]. Similar conclusions were reached by Zimmermann et al. [123], who examined the effectiveness of virtual reality versus traditional presentations in mass environmental campaigns. They found that virtual campaigns exert a stronger influence on their audiences.

In recent years, numerous studies have presented various scenarios with differing levels of realism to investigate the effectiveness of climate change education. In the research conducted by Thoma et al. [14], different visualizations of the Aletsch Glacier were created. This is the largest and most renowned glacier in Switzerland, which, as a result of climate change, may shrink significantly within the next 80–100 years. Participants were shown a simulation of glacier melting over a few minutes with varying levels of visual realism. By manipulating parameters such as texture resolution and repetition, glacier topography, atmospheric perspective, and colour correction, three conditions were created: a well-designed abstract condition, a well-designed realistic condition, and a less sophisticated realistic condition. A total of 142 participants (82 women, 60 men) took part. The study demonstrated that both the New Ecological Paradigm (a widely used measure of pro-environmental orientation and environmental concern) and connectedness with nature (a key component of ecological awareness) increased significantly compared to the control group (video) across all three immersive VR conditions.

Preparing scenarios and executing them in the right quality requires not only high-end equipment, but also a knowledge of basic psychology. The image presented, together with sound and opportunities for active participation in the scenario, should be based on appropriate psychological reactions in people, including both emotional and cognitive reactions. Then, the educational effect associated with the need to apply the principles of sustainable development on a daily basis and the understanding of phenomena and processes occurring in nature will be greatest [123,124].

The use of VR often involves not only 360° cameras, but also photogrammetry or LIDAR scanners to reproduce reality as accurately as possible [54,115,123]. This material is necessary for further work involving the preparation of a model of spatial and biological changes resulting from climate change and environmental pollution. High-quality sound recorders allow for very realistic sounds to be played back through headphones during the presentation of the scenario. The image combined with sound allows for a better experience in the virtual world [125]. The use of head-mounted displays, such as the HTC Vive, which allow the user to view the space around them, as well as other sensors that make virtual reality more intense for users, intensifies the effect of the content contained in the message. Motion sensors are often used to measure human movements and adjust the displayed image to the user’s position. Controllers that allow actions to be performed in virtual reality increase our involvement in the presented scenario. As a result, the content is perceived more intensely by the recipient and has a better and longer-lasting effect [118,126]. When the recipients are adults, it is also worth monitoring their physiological functions, such as changes in heart rate (HRV), skin conductance sensors (GSR), heart rate sensors, blood pressure sensors, and respiration sensors. The use of highly sensitive psychophysiological sensors allows for an objective assessment of the response to the content conveyed in the scenario [127]. However, it should be remembered that interpreting the results obtained is not easy and requires considerable experience. An electroencephalogram (EEG) may also be useful. Measuring brain activity can tell us a lot about the impact of the scenario content on the recipient [128].

When studying educational effects, measuring the increase in knowledge is not a major problem, but assessing the extent to which this knowledge has influenced our behaviour is, especially over time. By using sensors during a VR session, we can easily examine the participant’s reaction. However, to verify the effectiveness of an educational method, it would be necessary to compare the results of the psychophysiological reactions obtained with periodic studies on changes or the persistence of specific behavioural trends in relation to the subject under study. For this purpose, periodic surveys can be conducted.

Environmental education is also strongly supported by games designed to raise players’ awareness of environmental protection and the negative changes caused by various human activities [129]. Such games may not only address climate change directly but also urban design (urban planning) aligned with the principles of sustainable development [130]. Fricker and Weidhaas [131] explored the use of VR in architectural design, showing how future buildings could be designed to adapt to their environment, making architecture more sustainable and resilient to climate change. Similar research in the context of infrastructure planning was conducted by Ismael [126], who examined whether VR technology, combined with engineering decision-support tools, can enhance engineers’ ability to visualize the long-term resilience of their projects to environmental change.

As in the case of environmental education, when using VR to design sustainable spaces, including climate-resilient buildings, it is important to reproduce real spaces or their elements as accurately as possible, which we will then modify within virtual reality. In the case of participant interaction with the scenario, sensors that increase the quality and possibilities of activities performed by the participant in virtual reality are also important [51,52,53,126]. In addition, research conducted by Alsswey et al. [132] has shown that eye-tracking technology in virtual reality allows us to study visual patterns in psychology and neuroscience and to examine the impact of architectural design elements on humans. On the other hand, heart rate variability and electroencephalography (EEG) measurements can contribute to the assessment of stress levels depending on landscape characteristics [128].

Among human activities, agriculture has the greatest negative impact on climate change. In order to effectively educate future generations in sustainable farming, VR-based training is increasingly being introduced in this area. Traditional agricultural training requires significant human resources (instructors), is time-consuming, and is often limited by geographic accessibility. Therefore, the possibility of VR-based training, including remote training, allows broader reach while achieving higher knowledge retention compared to traditional text-based methods [133].

Virtual reality can also be used in less obvious ways in environmental protection. Wang et al. [134] employed VR to present four models of street lighting. Urban lighting has become an increasingly discussed issue due to its negative effects on both the environment and human health. Excessive night-time lighting, known as light pollution, is now widely recognized as a serious problem. Over-illumination disrupts bird behaviour (e.g., orientation disturbances, changes in day-night cycles) and adversely affects human health, including sleep disorders that may lead to broader health problems. Furthermore, it significantly increases energy consumption, often supplied through conventional methods. The authors [134,135] developed lighting scenarios aimed at ensuring pedestrian safety while reducing environmental impact. Participants were able to evaluate the proposed systems. The findings may be used in the design of lighting systems that both meet user requirements and reduce energy consumption, thereby minimizing negative environmental impacts. The use of psychophysiological sensors in such studies would not only reveal the subjective feelings of respondents regarding their sense of safety in different lighting conditions in public spaces, but would also allow for an objective assessment. This is important when designing for a larger audience, as the sense of safety is a very individual matter that is difficult to verify. Eye-tracking sensors may also be useful here, as they provide information about areas requiring special attention when designing lighting, or facial expressions, which are an indirect measure of stress (facial muscle tension) [132,136].

Another example of a less obvious use of VR in environmental protection is sustainable tourism. Alonso-Garcia et al. [137] conducted research on the potential of VR in tourism activities to support the implementation of the 17 Sustainable Development Goals. They concluded that VR can serve as a substitute for physical tourism. Virtual tours may become more popular in the near future. Not only would this be a much cheaper form of tourism, but it is also in line with the principles of sustainable development, as we do not burden the environment with pollution from means of transport [138,139]. However, for them to be attractive, the quality of the images and sounds presented, if they are required for the full reception of the tourist attraction, is extremely important. The simultaneous use of eye tracking and facial expression sensors will allow the identification of areas requiring improvement in the quality of the scenario/image of the objects, which can significantly increase their attractiveness for virtual tourists.

### 3.4. VR in Occupational Safety

In the context of occupational safety, VR is gaining importance as an educational, diagnostic, and simulation tool [2]. It enables the delivery of training under highly realistic conditions without exposing workers to actual hazards. The technology allows for interactive exercises related to evacuation, machine operation, or identification of potential workplace risks. Equipped with VR headsets and appropriate controllers, users can interact with the environment, which translates into better retention of procedures and higher engagement in the learning process. VR also facilitates real-time assessment of participants’ progress and errors, which can help diagnose competency gaps. Moreover, this technology is applied not only in initial training but also in periodic refresher courses, audits, and accident analysis through virtual reconstruction. As a result, VR constitutes a key component of the digital transformation of workplaces and vocational education, aligning with broader Industry 4.0 and organizational sustainability trends.

A review of selected scientific publications indicates that the application of VR in occupational safety training is expanding and proving effective across various industrial sectors. Growing empirical evidence confirms that VR not only enhances the efficiency of knowledge transfer but also supports the development of behavioural and organizational competencies related to safety. For instance, studies in cardiology laboratories demonstrated that VR can effectively support training in radiation safety by providing realistic, repeatable, and measurable scenarios without exposing staff to actual risks [140]. In the photovoltaic sector, VR has been employed as an educational tool supporting sustainable development goals while better preparing workers for high-risk environments [141].

In construction, VR has found particularly broad application, including training on working at heights, drone operations [142,143], and heavy machinery handling. Furthermore, it is used in ergonomics and occupational rehabilitation, assisting in the assessment of postural risks and the design of safer workplaces [144,145].

The literature also highlights VR’s impact on improving risk perception, strengthening safety culture, and streamlining inspection and control processes [146,147]. In recent years, approaches based on 360° techniques and narrative storytelling in VR environments have gained importance, enhancing immersion and cognitive engagement during training [148]. Additionally, research suggests that VR can not only support technical training but also foster proactive safety behaviours and even reduce occupational stress, particularly in healthcare and mining industries [111]. Such broad and diverse applications underscore VR’s growing role as an innovative and effective component of safety education in the Industry 4.0 era.

A key advantage of VR technology is its ability to create repeatable and individually tailored training scenarios that reflect specific working conditions, industry procedures, and potential hazards. This adaptability makes VR highly suitable for modern industries, especially high-risk sectors such as construction, chemical, mining, and energy industries.

Scorgie et al. [28] note that fewer than 10% of publications are grounded in educational theories, underlining the need for further research. Nevertheless, Strzałkowski et al. [2] showed that VR can be successfully applied in occupational safety training in mining and construction. Toyoda et al. [29] highlight the superiority of fully immersive VR systems over conventional computer-based training programs. Similarly, Bęś and Strzałkowski [16] and Namkoong et al. [149] provide empirical evidence of VR’s effectiveness in changing safety behaviours and improving users’ safety knowledge and skills.

The development of VR technology opens new possibilities in occupational safety, with its full potential realized through integration with other digital technologies such as augmented reality (AR) and digital twins [150,151]. Combining VR with AR enables the creation of integrated, immersive simulation environments that not only replicate real conditions but also respond in real time to user actions, enhancing training effectiveness in evacuation, working at heights, or machine operation [152,153]. AR allows contextual information to be overlaid on real-world objects, facilitating faster and more accurate decision-making during operations in challenging conditions [153,154]. In sectors such as mining and construction, characterized by variability and high risk, VR integrated with spatial data (e.g., geodetic 3D models) and IoT sensor data enables real-time monitoring of working conditions and early detection of potential hazards [155]. The use of digital twins allows for the simulation of entire technological processes or industrial facilities in real time, incorporating operational data to support training and emergency scenario analysis [2,155,156].

XR technologies (VR and AR) are also becoming valuable tools for supporting workplace ergonomics and identifying safety-critical points. XR systems can provide “field support” through real-time remote expert consultations, reducing response times and improving the quality of interventions without requiring physical presence [157,158].

Although numerous studies demonstrate the pedagogical and organizational benefits of VR-based safety training, several technical challenges and performance bottlenecks still constrain its broader adoption in occupational contexts. One recurring issue is the latency and synchronization delay between motion-tracking sensors, haptic controllers, and rendering systems, which can disrupt immersion and cause sensory dissonance during complex training tasks. Similarly, limitations in sensor precision—for instance, IMU drift during repetitive movements or poor GNSS signal reception in enclosed industrial environments—may reduce spatial accuracy and the realism of simulated scenarios. Another difficulty lies in the fusion of heterogeneous sensor data (e.g., motion, physiological, and environmental inputs), which often requires advanced calibration and filtering algorithms to avoid conflicting measurements or frame-rate instability. Different technical approaches also exhibit distinct strengths and weaknesses. 360° video-based VR provides high visual realism and low hardware requirements but lacks interactivity and user feedback. Conversely, fully immersive, sensor-driven VR systems offer dynamic interaction and objective data collection (e.g., gaze or motion tracking) but demand expensive equipment and complex data processing pipelines. Hybrid XR solutions combining VR with AR and IoT sensors show promise for real-time situational awareness, yet they remain vulnerable to latency and connectivity issues, especially in field conditions.

Overall, the main technical barriers identified across case studies include high computational demand, sensor misalignment, inconsistent data quality, and the absence of universal interoperability standards. Addressing these issues—through improved sensor fusion frameworks, real-time error correction, and standardized protocols—will be essential for transitioning current VR safety systems from experimental prototypes to robust, scalable industrial applications [4,28,159,160].

Several commercial implementations of VR-based occupational safety training have been successfully adopted across high-risk industries such as construction and mining. Companies like Immersive Technologies (mining sector), Another Reality Studio (construction), and Program-Ace (VR training developers) have developed industry-grade simulators that allow employees to practice equipment operation, hazard identification, and emergency responses in immersive, risk-free environments. These platforms are often built upon research findings from earlier academic studies, reflecting the transfer of VR concepts from experimental settings to operational industry tools [2,28,161]. For instance, Immersive Technologies’ WorksiteVR™ simulators are based on real-world mining scenarios and have been shown to improve operator performance, decision-making, and adherence to safety protocols. Similarly, the VR construction training project by Another Reality Studio for a Fortune 500 company illustrates how research on risk perception, immersion, and learning transfer is informing corporate training programs. These cases demonstrate how scientific advances in VR-based safety education have evolved into practical, industry-ready applications, reinforcing the relevance and applicability of ongoing academic work in this field.

Despite the promising results and growing body of evidence supporting the effectiveness of VR-based occupational safety training, several critical research gaps remain. Most existing studies focus on short-term learning outcomes and self-reported measures rather than on long-term behavioural change and skill retention [2,28]. Furthermore, only a limited number of works apply established educational and cognitive learning theories, such as experiential learning, constructivism, or cognitive load theory, to explain the mechanisms underlying training effectiveness. This theoretical underrepresentation restricts the transferability of results and hinders the development of standardized evaluation frameworks [16,162]. Another major limitation is the insufficient integration of physiological and behavioural sensors (e.g., heart rate, gaze, or motion tracking) that could provide objective metrics for assessing user engagement, stress, and situational awareness. Future research should therefore focus on longitudinal, theory-driven studies combining quantitative sensor data with qualitative behavioural assessment, enabling a more comprehensive evaluation of how immersive safety training influences real-world performance and safety culture [163].

## 4. Limitations and Potential of Virtual Reality Technology

### 4.1. Limitations of VR Technology

Engineering, as both a scientific and practical discipline, plays a key role in the development of the modern world by integrating different fields of knowledge and technology. Its purpose is to apply scientific and technological principles to the creation, design, and maintenance of systems, processes, and products that meet specific human requirements and needs. In recent decades, engineering has not only evolved but also adopted new directions, particularly in response to technological progress that is transforming the way work is performed.

The introduction of virtual reality (VR) has significantly revolutionized how engineers and specialists in related fields are trained and perform their work. The possibility of using VR as a ground-breaking technology in recent years represents a clear example of engineering applied in practice. VR, a technology that enables the creation of immersive experiences in computer-generated environments, has gained popularity across multiple sectors, as discussed earlier. Nevertheless, despite its many potential applications, limitations associated with VR implementation and usage can be observed. These challenges represent critical areas for further technological development and can be grouped into several main categories: technological issues, lack of qualified personnel, health concerns, resistance to change, ethical considerations, and insufficient funding.

A major challenge is the presence of technological barriers. Many organizations face difficulties in implementing complex VR technologies, including usability and functionality issues [164]. VR requires advanced hardware and software, which are not always accessible or sufficient for effective use [2,16,165]. According to Trifu et al. [166], high-quality VR hardware, such as ergonomic headsets and reliable control systems, is essential to achieve immersive experiences, which form the foundation of VR functionality. However, technological challenges related to installation, configuration, and maintenance can discourage adoption [164]. Latency between user movements and the image displayed in VR headsets is another recurring issue, reducing immersion and potentially causing discomfort [167].

The complexity of VR application design represents another significant obstacle, requiring the close collaboration of experts from multiple disciplines. Effective development should be guided by well-defined design concepts and project objectives. Yet, long and costly project cycles often hinder the timely adaptation of VR applications to evolving user needs. Moreover, some VR applications fail to accurately replicate real-world conditions, particularly in relation to unpredictable environmental factors. Despite its advanced nature, VR frequently relies on simplified environmental models, resulting in discrepancies between simulations and reality [108,110]. Furthermore, certain working environments, such as underground mines, limit the direct applicability of VR. Issues with connectivity, workspace constraints, or environmental restrictions reduce the feasibility of in situ VR deployment [100,107,110]. As a result, many VR solutions are primarily used for planning, training, or simulation rather than in real operational environments. Another technological limitation concerns the lack of standardized software and technical protocols, which contributes to market fragmentation and limited interoperability across VR systems. Since developers often implement unique specifications for interfaces and functionalities, applications are not easily transferable between devices [167].

Another major barrier is the lack of qualified personnel. Without well-trained staff and access to educational resources, the use of VR in organizations remains limited. Employees must not only understand how the technology operates but also be capable of adapting it to user-specific requirements. Research by Hoang et al. [168] demonstrated that the educational background and organizational maturity of companies significantly influence the acceptance of VR as a practical tool.

Health-related issues also present a considerable challenge. Symptoms of cybersickness, such as dizziness and discomfort, reduce users’ ability to engage with VR systems [167,169,170]. Cybersickness can be caused by prolonged exposure, predisposition to motion sickness, fatigue, postural factors, or difficulties in adapting to VR applications [167,171]. In particular, inexperienced users often report strong discomfort, which discourages long-term adoption of the technology.

Resistance to change, both internal and external, is another important challenge. Human behaviour is often characterized by reluctance toward unfamiliar technologies [168]. In the case of VR, many organizations struggle with insufficient awareness of the potential benefits and concerns about implementation costs [172]. High initial investment and ongoing expenditures may lead to scepticism among decision-makers [173].

The deployment of VR across various domains also raises ethical issues requiring careful consideration [174]. Data privacy is one of the most critical concerns. Users are often required to provide personal information, raising questions about how this data is stored, used, and potentially shared with third parties. Improper handling of such data may result in breaches of user privacy [175]. Emphasizing transparency in data collection and management reflects the importance of building trust between users and technology providers.

Finally, insufficient funding restricts the ability to design scenarios and implement VR-based solutions. The financial position of organizations adopting VR frequently determines the success or failure of its application [176]. Consequently, funding programs and grants are necessary to support institutions in obtaining the resources required to fully leverage this innovative technology.

### 4.2. Sensor Technologies as Enablers of Immersive VR Applications

Sensor technologies play a fundamental role in enabling the immersion, interactivity, and realism of virtual reality (VR) applications in engineering contexts. In practice, the effectiveness of VR-based solutions relies heavily on the accuracy, responsiveness, and integration of various sensors. Among the most critical components are inertial measurement units (IMUs), which are responsible for motion tracking and orientation detection in VR headsets and controllers. These sensors allow users to interact naturally with virtual environments by synchronizing physical movements with digital feedback. For example, in geodetic and mining simulations, IMUs support precise gesture recognition and spatial navigation, enhancing the realism of tasks such as surveying or equipment operation [177,178].

Another vital group of sensors includes Global Navigation Satellite System (GNSS) modules, particularly when VR applications incorporate georeferenced spatial data or require real-world positioning. In geomatics, GNSS-based data are commonly used for aligning photogrammetric or LiDAR-derived models, which are later visualized in VR. GNSS integration also facilitates the development of VR digital twins, in which real-time positioning data enrich scenario realism and analytical value [179,180].

In the domain of occupational safety and training, physiological monitoring sensors—such as heart rate monitors, electrodermal activity sensors, or EEG—are gaining importance. These sensors allow for real-time feedback on users’ stress levels, engagement, and cognitive load, enabling adaptive training scenarios that respond to the user’s physical or mental state. They are especially valuable in high-risk environments like mining or heavy industry, where monitoring trainee responses can improve hazard recognition and training effectiveness [181].

Additionally, environmental sensors (e.g., air quality, temperature, lighting) are being integrated with VR to simulate environmental conditions more accurately or to collect contextual data during field operations. In environmental protection and urban planning scenarios, VR environments powered by real sensor inputs enable users to experience and assess simulated pollution levels, thermal comfort zones, or light pollution effects [182].

The growing trend toward sensor fusion—the integration of multiple types of sensors (e.g., IMU + GNSS + RGB-D cameras)—allows for higher fidelity, reduced latency, and enhanced realism in VR environments. This fusion is particularly relevant for mobile mapping, wearable interfaces, and dynamic simulations used in engineering practice [179,180].

Future VR developments are expected to rely increasingly on wearable and IoT-based sensor systems that provide context-aware, real-time data. These advances will support not only greater immersion but also improved decision-making, personalization, and safety monitoring across diverse engineering applications.

### 4.3. The Future of VR Technology and Further Research Directions

Virtual reality (VR) is a technology that has evolved significantly in recent years, and its future appears highly promising. Key research directions in this field demonstrate that VR improves business decision-making, enhances learning efficiency, and transforms user experiences by enabling immersive and active access to diverse content. Similarly, the future of VR technology in engineering is increasingly innovative, shaping a new paradigm in technical education and industrial engineering processes. Studies indicate the dynamic development of VR, which has become an essential tool in engineering education by providing immersive experiences that support a deeper understanding of complex technical concepts [6,183].

The application of VR in engineering is becoming a fundamental aspect of design and simulation, allowing engineers to model and test complex systems in virtual environments, thereby significantly reducing the costs and risks associated with real-world experimentation [4]. In practice, using VR for process visualization can increase both efficiency and precision, particularly in environments that are difficult to replicate physically [183]. Moreover, educational tools such as simulations provide young engineers with hands-on experience that strengthens knowledge acquisition, which is particularly important in the context of engineering applications [184].

The role of VR in engineering is further strengthened through integration with augmented reality (AR) and artificial intelligence (AI). By synchronizing these technologies, engineers can support decision-making based on real-time data, ensuring even greater precision in projects [6]. For example, combining VR simulations with AR provides engineers with unique opportunities to interact with three-dimensional models, merging visual perception with practical skills [12]. The new possibilities created by VR and AR also enable flexible approaches to engineering education, tailored to the individual needs of students. Learners can develop skills at any time and place, addressing the growing demands of modern educational practices [2,6,16]. Additionally, virtual laboratories allow students to perform simulations of complex processes, offering not only an attractive but also an educationally effective form of training [185]. Research shows that such interactive approaches can significantly increase learning effectiveness, especially in technical and engineering disciplines where understanding complex systems is crucial [186]. It is also important to note that engineering, including its VR applications, plays a vital role in modern social transformations, promoting the integration of diverse groups, including individuals with special needs.

Another growing area of VR application in engineering is the integration of digital twin systems in industrial environments. Digital twins allow for the simulation of real production processes, enabling process management and operational optimization. Such solutions are particularly significant for industry, where precise digital models can establish new standards of quality and efficiency. The integration of VR with digital twins enables more effective training and real-time monitoring of production processes. Research on the convergence of VR and digital twins is opening a new era in industrial automation and digitalization [187,188,189,190,191].

The future of VR is closely linked to the development of network infrastructure, including the implementation of 5G and upcoming 6G standards, which allow for the transmission of massive amounts of data in real time. High-bandwidth connections ensure minimal latency, which is crucial for VR applications requiring seamless interaction. Moreover, advances in cloud architectures and parallel processing systems will enable remote rendering of complex scenes, opening new opportunities for mobile VR applications. Integrated communication systems will support the synchronization of multiple users within a single virtual environment, fostering the growth of online communities. As a result, future VR systems will be able to fully leverage global networks to generate comprehensive, interactive experiences [192,193].

From a technological development perspective, the future of VR in engineering fields is moving toward the integration of virtual and real user experiences. Such approaches can lead to more holistic teaching, deeper understanding of processes, and more effective knowledge acquisition. As the literature demonstrates, real-world experiences enriched by VR foster critical reflection on engineering processes by engaging multiple human senses [194,195]. Another crucial aspect of VR’s future is the advancement of haptic interfaces and direct manipulation technologies, which allow users to interact more naturally with virtual environments. These systems combine advanced touch and force-sensing technologies, enabling more realistic tactile feedback [196,197]. Research on direct manipulation of digital objects is also leading to the development of new methods of user-system interaction that may greatly enhance work efficiency in VR [197]. The integration of haptic technology with visual systems allows for more comprehensive applications of VR in training and educational simulations. This approach promotes the development of intuitive interfaces, reducing barriers between the user and the virtual environment [196,197]. In the future, further research into direct manipulation will be critical for advancing educational, training, and entertainment applications.

Research on perceptual and sensorimotor adaptation in VR focuses on how users behave in virtual environments. Adaptive processes are analyzed using advanced measurement techniques, allowing for the design of individual response patterns. Understanding how the human nervous system operates makes it possible to introduce modifications into VR applications [198,199]. Future studies will rely on advanced sensorimotor tools, which are crucial for creating more natural VR experiences.

Cognitive data in VR, processed through machine learning algorithms and AI, are becoming a central research direction, aiming to tailor environments to individual user needs. These systems analyze user behaviour and enable the development of scenarios based on recipient responses, resulting in more detailed and engaging VR applications. Research in this area also includes adaptive predictive models that can anticipate user behaviour based on behavioural data analysis. Furthermore, through data analysis algorithms, VR systems are capable of real-time learning and updating [190,191]. The integration of AI and VR also opens possibilities for automation and personalization of VR applications. Further research in this field has the potential to revolutionize the way users interact with digital environments.

It is also important to develop certification and validation systems for VR applications, which would allow for broader use in educational and vocational adaptation processes. Another area requiring deeper exploration is the long-term effectiveness of VR in knowledge retention. Most existing research is limited to short-term measurements taken immediately after VR use, while there is a lack of meta-analyses examining long-term behavioural changes among users.

The development of VR technology is inevitable, and its integration into various engineering domains will reshape educational and industrial experiences, fostering collaboration and innovation [200]. The future of engineering with VR makes this field one of the most exciting directions for students and professionals eager to engage with a rapidly evolving technological landscape. In the coming years, the use of VR in engineering is expected to increase significantly, transforming the way engineers design, test, and implement innovations. This technology will influence the growth of technical sciences. The use of VR in design and simulation is becoming a standard, and it is anticipated to enable more efficient learning and the implementation of increasingly complex projects in shorter timeframes [4,183,201].

## 5. Conclusions

Virtual reality technology represents one of the most dynamically developing tools supporting engineering activities. Its application in areas such as geodesy and geomatics, mining, environmental protection, and occupational safety enables the optimization of design processes, improvement of training efficiency, and enhancement of quality and safety in operations. Through realistic simulations, VR allows users to be immersed in conditions difficult to reproduce in reality, thereby creating new practical, research, and educational perspectives. Additionally, the integration of various sensors facilitates enhanced representation of reality in immersive environments, thereby enabling behavioural research during utilization. In summary, the key areas of VR use in combination with sensors are presented in Table 2.

In the context of geodesy and geomatics, VR technology facilitates the visualization of spatial data. Examples of applications include the creation of three-dimensional terrain models and the analysis of geodetic data in virtual environments, which supports decision-making in spatial planning and resource management. Moreover, VR serves as an educational tool, allowing students and professionals to practice the use of surveying instruments and geoinformatics analyses in virtual conditions. Such innovations contribute to improving efficiency and accuracy in the execution of geodetic and geomatics projects, minimizing the risk of errors arising from misinterpretation of data.

In mining, VR technology enables realistic representation of mining objects (workings, spoil tips, technical infrastructure), supporting the processes of excavation design, extraction process simulation, and safety analysis. Emergency simulations are of particular importance, as they allow workers to be trained in crisis response without endangering their health. Consequently, VR contributes to improving workplace safety and reducing accident risks in an industry especially exposed to numerous hazards.

In environmental protection, VR serves as an effective tool for education and ecological awareness. Through simulations, it is possible to visualize the effects of water and air pollution or deforestation. This facilitates a better understanding of ecological processes and highlights the consequences of human activity. Virtual reality also supports the analysis of industrial impacts on the environment, for example by simulating pollutant emissions, noise, or landscape changes. Virtual models assist in public consultations by enabling visualization of the effects of planned actions and increasing ecological awareness. Additionally, VR acts as an educational tool, allowing users to better understand mechanisms of environmental degradation and methods of counteracting these processes.

In the area of occupational safety, virtual reality offers the possibility of creating realistic training programs in a safe environment. Workers can learn how to operate machinery, respond to accidents, or apply evacuation procedures under simulated conditions. Training conducted with VR can significantly enhance participants’ skills and improve learning outcomes compared to traditional training methods. Furthermore, VR technology can be used to assess and improve workplace ergonomics, thus increasing comfort and reducing the risk of accidents caused by inappropriate conditions.

Virtual reality is an innovative tool with vast potential across numerous engineering domains. It enables better understanding of complex phenomena, raises safety standards, supports sustainable development, and accelerates decision-making processes. Prospects for the development of VR indicate its continued evolution, taking into account technological advancements and behavioural factors. The parametric development of VR and its integration with other digital solutions may revolutionize engineering practices across industries in the future. Despite certain challenges and barriers, VR is becoming an indispensable element of modern engineering approaches. The integration of VR with traditional work methods transforms engineering processes, making them more intuitive, safe, and precise.

## Figures and Tables

**Table 1:** Keywords and search results.

Industry	Keywords	Search Results	Selected Results
Geodesy and Geomatics	VR OR virtual reality OR geomatics OR land surveying OR geodesy OR surveying instruments OR field measurements OR total station OR GNSS OR cartography OR GIS	2461	881
Mining	virtual reality OR vr AND mining engineering OR mining industry OR mining operation”	161	84
Environmental protection	vr OR virtual reality AND ecological education OR climate change OR environmental protection	526	348
Occupational safety	VR OR virtual reality AND occupational safety OR safety at work OR occupational health and safety OR OHS OR OSH	184	82

**Table 2:** Main areas of application for VR technology.

Engineering Area	Main Application Scenarios	Core Sensor Types Relied upon	Key Technical Challenges	Future Sensor-Driven Directions
Geodesy and Geomatics	Training and simulation of surveying procedures/measurements for example levelling, GNSS, total station setup	motion capture systems, head movement sensors, 360° cameras, surveying instrument model	Realistic replication of field conditions	Development of training laboratories connected with sensors, attractiveness of training courses for users and feedback mechanisms
Geodesy and Geomatics	3D visualization and analysis of spatial data	head movement sensors, 360° cameras, integration with Augmented Reality sensors	Integration of heterogeneous datasets, data volume and GPU constraints, loss of accuracy during mesh decimation, processing	Automation of data fusion and optimization of rendering workflows
Geodesy and Geomatics	Cultural heritage documentation and landscape reconstruction (visualization of archaeological or degraded sites)	motion capture systems, head movement sensors, 360° cameras	Balancing visual realism and metric precision and texture optimization	High-fidelity 3D scanning and AI-driven model reconstruction
Mining	Immersive training for drilling, blasting, equipment operation, and emergency evacuation procedures	IMU, motion-tracking sensors, physiological sensors (heart rate), GNSS	Sensor latency and drift affecting synchronization between user motion and simulated environment; limited realism in haptic feedback	Integration of multimodal sensor data for real-time feedback; adaptive training environments using AI-driven performance monitoring
Mining	Real-time visualization of underground conditions for safety assessment and ventilation management	laser tracking, temperature and humidity sensors, IoT nodes	Data fusion from heterogeneous sensors in harsh environments; unstable wireless connectivity underground	Development of digital twins integrating geospatial and sensor data; improved IoT-VR interoperability standards
Mining	3D visualization and virtual prototyping of mine layouts, geological structures, and operational processes	laser tracking, photogrammetry, UAV-based imaging, GNSS	Large-scale spatial data processing; alignment errors between datasets; limited rendering capacity for complex geological models	Enhanced real-time 3D reconstruction; integration of AR/VR with BIM and GIS systems for collaborative mine planning
Environmental protection	Educational scenarios of climate change effects taking into account temporal and spatial distance	IMU, motion capture systems, 360° cameras, heart rate monitors, skin conductivity sensors, EEG	realistic image rendering for climate change in landscapes/urban spaces/ecosystems	assessment of cognitive and behavioural responses to presented information and images, supported by AI
Environmental protection	Designing urban spaces in accordance with the principles of sustainable development—visualization of possible effects	Eye-tracking, hand gesture tracking, head movement sensors	realistic representation of urban spaces and the changes introduced in them, together with a presentation of their effects in the field of urban planning	assessment of the attractiveness of new projects and proposed changes in urban space supported sensors and real-time adaptation of VR content
Environmental protection	Virtual training courses on sustainable agriculture	motion capture systems, 360° cameras, heart rate monitors, skin conductivity sensors, EEG	realistic representation of aspects related to sustainable agriculture	AI- and biofeedback-supported training content adapted in real time to the personalized needs of the trainee
Occupational safety	Interactive training in evacuation, hazard recognition, and equipment operation	IMU, motion capture systems, 360° cameras	Sensor latency, drift, and occlusion in confined environments	Sensor-based real-time adaptation of VR content during procedural safety training
Occupational safety	Competency diagnostics and behavioural assessment using immersive VR scenarios	Eye-tracking, hand gesture tracking, head movement sensors	Lack of standardized evaluation metrics and data interpretation frameworks	AI-supported evaluation of cognitive and behavioural responses to simulated hazards
Occupational safety	Physiological monitoring during stress-inducing or fatigue-relevant training	Heart rate monitors, skin conductivity sensors, EEG	Limited physiological data integration and variability across individuals	Theory-driven, biofeedback-enhanced VR training for personalized safety learning

## Data Availability

No new data were created or analyzed in this study. Data sharing is not applicable to this article.
